# Practitioner's Bookshelf

**Published:** 1892-09-17

**Authors:** 


### PRACTITIONERS' BOOKSHELF.

### CARDIAC OUTLINES.*

The author informs us in the preface that the publication of this collection of cardiac outlines was undertaken with a threefold object ; to help the beginner through his first difficulties, to encourage the clinical clerk in the cultivation of the graphic method as a means to thoroughness and accuracy, and to place at the disposal of the practitioner an easy method of adequately recording important clinical observations. The little book does not profess to be a treatise on cardiac diseases ; the author has aimed at providing the reader with a method which he may find equal to the requirements of cardiac study. No student can read this book carefully and apply the directions given at the bedside without obtaining a very accurate idea of the anatomical relations of the heart. The accurate outlining of the heart is a good and useful practice for medical students, but we are bound to confess that to expect a medical student to provide himselff with a separate book on the physical investigation of all the equally important viscera is a grand mistake. While this work on the heart's anatomy will be most acceptable to the teacher, we believe that it will only tend to unduly exaggerate the importance of mere physical signs in the mind of a medical student.

* "Cardiac Outlines for Clinical Clerks and Practitioners," by William Ewart, M.D., F.R.C.P.; pp. 165, with sixty-two illustrations : (London Baillière, Tyndall, and Cox).

